# Methyl-donor depletion of head and neck cancer cells in vitro establishes a less aggressive tumour cell phenotype

**DOI:** 10.1007/s00394-017-1411-5

**Published:** 2017-03-01

**Authors:** Vanessa Hearnden, Hilary J. Powers, Abeir Elmogassabi, Rosanna Lowe, Craig Murdoch

**Affiliations:** 1 Human Nutrition Unit, Department of Oncology, University of Sheffield, Sheffield, S10 2RX UK; 2 School of Clinical Dentistry, University of Sheffield, Sheffield, S10 2TA UK

**Keywords:** DNA methylation, Head and neck cancer, Methyl donor, Apoptosis, DAPK

## Abstract

**Purpose:**

DNA methylation plays a fundamental role in the epigenetic control of carcinogenesis and is, in part, influenced by the availability of methyl donors obtained from the diet. In this study, we developed an in-vitro model to investigate whether methyl donor depletion affects the phenotype and gene expression in head and neck squamous cell carcinoma (HNSCC) cells.

**Methods:**

HNSCC cell lines (UD-SCC2 and UPCI-SCC72) were cultured in medium deficient in methionine, folate, and choline or methyl donor complete medium. Cell doubling-time, proliferation, migration, and apoptosis were analysed. The effects of methyl donor depletion on enzymes controlling DNA methylation and the pro-apoptotic factors death-associated protein kinase-1 (DAPK1) and p53 upregulated modulator of apoptosis (PUMA) were examined by quantitative-PCR or immunoblotting.

**Results:**

HNSCC cells cultured in methyl donor deplete conditions showed significantly increased cell doubling times, reduced cell proliferation, impaired cell migration, and a dose-dependent increase in apoptosis when compared to cells cultured in complete medium. Methyl donor depletion significantly increased the gene expression of *DNMT3a* and *TET-1*, an effect that was reversed upon methyl donor repletion in UD-SCC2 cells. In addition, expression of *DAPK1* and *PUMA* was increased in UD-SCC2 cells cultured in methyl donor deplete compared to complete medium, possibly explaining the observed increase in apoptosis in these cells.

**Conclusion:**

Taken together, these data show that depleting HNSCC cells of methyl donors reduces the growth and mobility of HNSCC cells, while increasing rates of apoptosis, suggesting that a methyl donor depleted diet may significantly affect the growth of established HNSCC.

**Electronic supplementary material:**

The online version of this article (doi:10.1007/s00394-017-1411-5) contains supplementary material, which is available to authorized users.

## Introduction

It is estimated that there were 140,000 new cases and 63,500 deaths from head and neck squamous cell carcinoma (HNSCC) in Europe in 2012 [1]. The 5-year age-standardised relative survival rate was approximately 40% for Europe overall with survival rates lowest in Eastern European and highest in Central, Western, and Northern European counties for all sub-categories of HNSCC such as oral cavity, oropharyngeal, laryngeal, and hypopharyngeal cancer [2]. Head and neck squamous cell carcinoma (HNSCC) is frequently (54%) detected at an advanced stage and so often has a poor prognosis with less than 55% overall 5-year survival following diagnosis [2, 3]. HPV infection is responsible for virtually all cervical cancers and approximately 35% of diagnosed oropharyngeal cancers worldwide, although there is a significant region to region variation globally [4]. Chaturvedi et al. predict that the number of HPV-positive HNSCC cases will exceed cases of cervical cancer by 2020, demonstrating the urgent need for a better understanding of the disease process and potential prevention strategies [5].

Folate is an essential dietary component required for the synthesis of nucleotides and for the synthesis and repair of DNA. There are several mechanisms by which folate status affects cancer risk. Low folate status has been shown to cause uracil mis-incorporation into DNA [6], cytogenetic damage [7], and impaired DNA repair [8] all of which increase the risk of neoplastic change. Indeed, epidemiological studies have shown that low dietary folate intake is associated with an increased risk of HNSCC carcinogenesis [9–11]. Conversely, other studies have shown that a high folate status may drive the growth of established tumours [12, 13]. In addition, there is evidence that folate status affects HPV infection and behaviour. Studies by our group and others have shown that low folate status is associated with an increased risk of cervical HPV infection [14, 15] and cervical intraepithelial neoplasia or invasive cancer [15]. The direction of influence of folate status on HPV persistence is not clear [14, 15]. Recently, Xiao et al. showed that low folate conditions increased HPV integration into the keratinocyte host genome and reduced viral capsid production in human keratinocytes in vitro [16]. Folate and other diet-derived methyl donors (choline, betaine, methionine) may also influence cancer risk and progression through epigenetic effects. The epigenetic control of gene expression is regulated, in part, by the methylation of cytosine–guanine dinucleotides within gene promoter regions, and dysregulated methylation in the promoter sequences of tumour-suppressor or oncogenes can promote carcinogenesis in HNSCC [17, 18]. For the methylation of DNA, methyl groups are transferred from the ultimate methyl donor, *s*-adenosyl methionine (SAM), onto cytosine residues in DNA by DNA methyltransferases (DNMTs). Whilst the factors that regulate DNMTs are not well understood, there is evidence that methyl donor depletion may lower DNMT expression in cervical cancer cells enzymes. DNMT1 preferentially adds methyl groups to hemi-methylated DNA and is responsible for maintenance of DNA methylation following cell division [19]. DNMT3a and 3b add methyl groups to CpG sites and are required for de novo DNA methylation [20]. In contrast, Tet methylcytosine dioxygenase 1 has been implicated in demethylation of DNA [21]. Poomipark et al. recently demonstrated methyl donor status altered the expression of DNMTs in cervical cancer cells [22]. Appropriate gene methylation is important for the regulation of many processes, including those involved in cancer progression such as cell cycle control and apoptosis. In addition, cell stress due to lack of essential nutrients may also lead to increased expression of key pro-apoptotic genes such as death-associated protein kinase-1 (DAPK1) [23] and p53 upregulated modulator of apoptosis (PUMA) [24], and so alterations in methyl donor availability may have profound effects on cell behaviour.

Here, we have developed an in vitro model of methyl donor-deficient HNSCC cells, with particular emphasis on HPV-positive HNSCC. We show, for the first time, that methyl donor deficiency significantly alters the phenotype of HNSCC cells by decreasing their proliferation and migratory capacity whilst up-regulating the expression of pro-apoptotic genes and increasing levels of apoptosis.

## Materials and methods

### Cell culture and methyl donor depletion

UD-SCC2 (HPV-positive) [25] and UPCI-SCC72 (HPV-negative) [26] cells were cultured in RPMI medium supplemented with 10% (v/v) fetal bovine serum (FBS), 100 IU/ml penicillin, and 100 μg/ml streptomycin (Sigma, Poole, UK), and confirmed as HPV-16 positive by qPCR and p16 staining. In addition to measuring in the HNSCC cell lines used in the model, *DAPK1* promoter methylation was also measured in UPCI-SCC89, UPCI SCC152, UPCI SCC154 [26], and FaDu [27]; the cervical carcinoma cell lines HeLa [28] and SiHa [29]; the oral dysplastic epithelial cell line (DOK) [30]; and the basaloid squamous cell carcinoma cell line (PE/CA-PJ34, clone C12) [31]. All cells were cultured at 37 °C, 5% CO_2_ as per supplier instructions. All cell lines were verified using short tandem repeat (STR) analysis (Public Health England). RPMI cell culture medium contains methyl donors at the following concentrations: l-methionine 101 µmol/L, choline chloride 21.4 µmol/L, and folic acid 2.26 µmol/L; this was designated ‘complete medium (100%)’. RPMI medium containing no l-methionine, choline chloride, or folic acid (0% methyl donors) was custom-made by Gibco^®^ (customisation of #11875093) and then supplemented with 10% (v/v) FBS, 100 IU/mL penicillin, and 100 μg/mL streptomycin. Complete medium and 0% medium were mixed in appropriate ratios to produce media containing increasing amounts of methyl donors (e.g., 40, 20, 10, and 5%) of the complete medium. To avoid a metabolic shock response to depleted medium, cells were gradually depleted of methyl donors over time for 4 days. Cells were then cultured in the experimental methyl donor concentrations for 4 days prior to seeding the cells for the experiments and experiments were performed at the methyl donor concentrations as indicated. The concentration of methyl donors in FBS is minimal [32]; the same batch of FBS was used throughout. For repletion experiments, cells were returned to complete culture media (100%) after a total of 15 days in depleted conditions and analysed 72 h later.

### Measurement of methyl donors

As a marker of disturbance to the methylation cycle, extracellular homocysteine was measured using a high-performance liquid chromatography detection kit (Chromsystems, Gräfelfing, Germany). Cell culture medium was collected and centrifuged to remove cell debris before storage at −80 °C. Homocysteine concentration was normalised to cell number. Intracellular choline, betaine, and methionine concentrations were determined using isotope dilution liquid chromatography tandem mass spectrometry as previously described [33].

### RNA extraction and quantitative RT-PCR

Total RNA was isolated (Bioline, London, UK) and 700 ng reverse transcribed using High Capacity cDNA Reverse Transcription Kit with RNase Inhibitor. Quantitative PCR was performed using a 7900HT Fast Real-Time PCR System with thermal cycles of 50 °C (2 min) and 95 °C (10 min) followed by 40 cycles of 95 °C (15 s) and 60 °C (1 min). For *DNMT* detection the reaction mix consisted of 300 nM of both forward and reverse primers (Sigma, Poole, UK), 125 nM FAM-labelled probe specific to *DNMT 1, 3a* and *3b* [34], 2X TaqMan^®^ mastermix, 0.5 µL β-2-Microglobulin (β2M) reference control with VIC-reporter dye, and 35 ng cDNA. Inventoried TaqMan^®^ FAM-labelled probes were used to measure expression of *DAPK1* (Hs00234480_m1), TET1 (Hs00286756_m1) and PUMA (Hs00248075_m1). β-2-Microglobulin (Hs00984230_m1) with a VIC-reporter dye was used as a reference control gene. Relative change in gene expression was calculated using the 2^−ΔΔCt^ method.

### Cell migration

Cell migration was measured using the Oris™ cell migration assay (Platypus Technologies, Madison USA). Cells were seeded into 96-well plates and a circular exclusion zone was created using a stopper to prevent cell adherence in the centre of the well as per the manufacturer’s guidelines. Once adhered, cells were treated with 0.5 µg/mL mitomycin C (Sigma, Dorset, UK) for 4 h to inhibit cell division, and the stopper was removed to create an exclusion zone of 5.37 ± 0.05 mm^2^ that was imaged using a Spot™ USB camera (Spot Imaging Solutions, Michigan, USA) at baseline and following cell migration after 72 h.

### Cell counts and cell proliferation

UD-SCC2 and UPCI-SCC72 cells were detached from tissue culture plates at 24, 72, and 168 h after seeding and live cells counted on a haemocytometer using trypan blue exclusion. Eighteen counts were performed for each condition and each experiment was performed three times. Cell doubling time was calculated using Doubling Time software (http://www.doubling-time.com). Cell proliferation was measured using CellTrace^™^ CFSE Cell Proliferation Kit. Cell suspensions (1 × 10^6^ cells/mL) were incubated with 1 µM CSFE CellTrace^™^ in PBS/0.1% BSA for 10 min at 37 °C, quenched in cold media, incubated for 5 min on ice, washed twice in PBS/0.1% BSA, and seeded into 6-well plates. The following day, negative cell controls were incubated with mitomycin C (0.5 µg/mL) for 4 h at 37 °C to prevent cell division. At 72 and 168 h, cells were detached, washed, and fixed in 4% paraformaldehyde. Cell fluorescence intensity was analysed using a BD^™^ LSRII flow cytometer (BD Biosciences, Oxford, UK) and the proliferation index calculated as fluorescence intensity of mitomycin C-treated negative control cells/sample cell fluorescence intensity.

### Apoptosis

Apoptosis analysis was performed using the TACS Annexin V-FITC, Apoptosis Detection Kit, (Trevigen Inc. Gaithersburg, MD, USA). UD-SCC2 and UPCI-SCC72 cells were detached, washed, and incubated with FITC-labelled Annexin V and propidium iodine immediately prior to analysis. Cell fluorescence was analysed using a BD^™^ LSRII flow cytometer (BD Biosciences). Fluorescence gates were set at the beginning of the experiment using unlabelled and single labelled cells as well as positive controls for necrosis (1% saponin) and apoptosis (30% H_2_O_2_ for 10 min) (Supplementary Fig. 1). Cells were considered apoptotic if they showed positive staining for Annexin V, necrotic if they were positive for propidium iodine but not Annexin V and live if negative for both stains.

### Immunoblotting of death-associated protein kinase 1 (DAPK1)

Protein was extracted from cell pellets using ice-cold RIPA buffer with protease and phosphatase inhibitors (Roche Diagnostics Ltd., Sussex, UK), and protein concentration calculated using the bicinchoninic acid assay. Twenty micrograms of protein were separated using Novex^®^ 4–12% Tris–Glycine gels and transferred to a nitrocellulose membrane by semi-dry transfer. Membranes were blocked with 5% (w/v) BSA in Tris-buffered saline containing 0.05% (v/v) Tween-20 (TBST), and membranes were then incubated with antibodies directed to DAPK1 (clone55; 1:250 dilution; Sigma Aldrich, Poole, UK) or phosphorylated DAPK1 (pSer308; 1 µg/mL dilution; Novus Biologicals, Cambridge, UK) overnight at 4 °C or with anti-β-actin (1:30,000; Cell Signalling Technologies^®^, Danvers, MA) for 1 h at room temperature. Membranes were washed and incubated with anti-mouse IgG horseradish peroxidase-conjugated secondary antibody (1:3000 dilution; Cell Signalling Technologies^®^). All antibodies were diluted in 5% BSA/TBST. Immuno-reactive proteins were visualized using an enhanced chemiluminescent substrate for detection of horseradish peroxidase activity (Pierce^™^ ECL Western Blotting Substrate, ThermoFisher).

### Genomic DNA isolation, bisulfite modification of DNA, and quantitative methyl-specific PCR

Genomic DNA was extracted from a panel of cell lines (QIAamp^®^ DNA Mini kit, Qiagen) and 1 µg DNA was bisulphite treated using the EZ DNA Methylation^™^ kit (Zymo Research, Freiburg, Germany). Methyl-specific qPCR (qMSP) was performed to determine the presence of methylated *DAPK1* promoter. The reaction mixture comprised of: TaqMan^®^ Universal Mastermix II no UNG, 250 nM forward and reverse primers, 250 nM FAM-labelled probe with minor groove binding modification [35], and 500 ng bisulphite-treated DNA. β-actin was used as the reference control gene and amplified as follows: 1xTaqMan^®^ Universal Mastermix II no UNG, 900 nM forward and reverse primers, 250 nM VIC-labelled probe, and 100 ng bisulphite treated DNA. A fully methylated DNA control was used as a positive control (Zymo Research Europe, Freiburg, Germany). The following thermal profile was performed: 95 °C for 10 min, then 50 cycles of 95 °C (15 s), 65 °C (5 s), and 62.5 °C (55 s) using a 7900HT Fast Real-Time PCR System.

### Statistical analysis

**Fig. 1:**
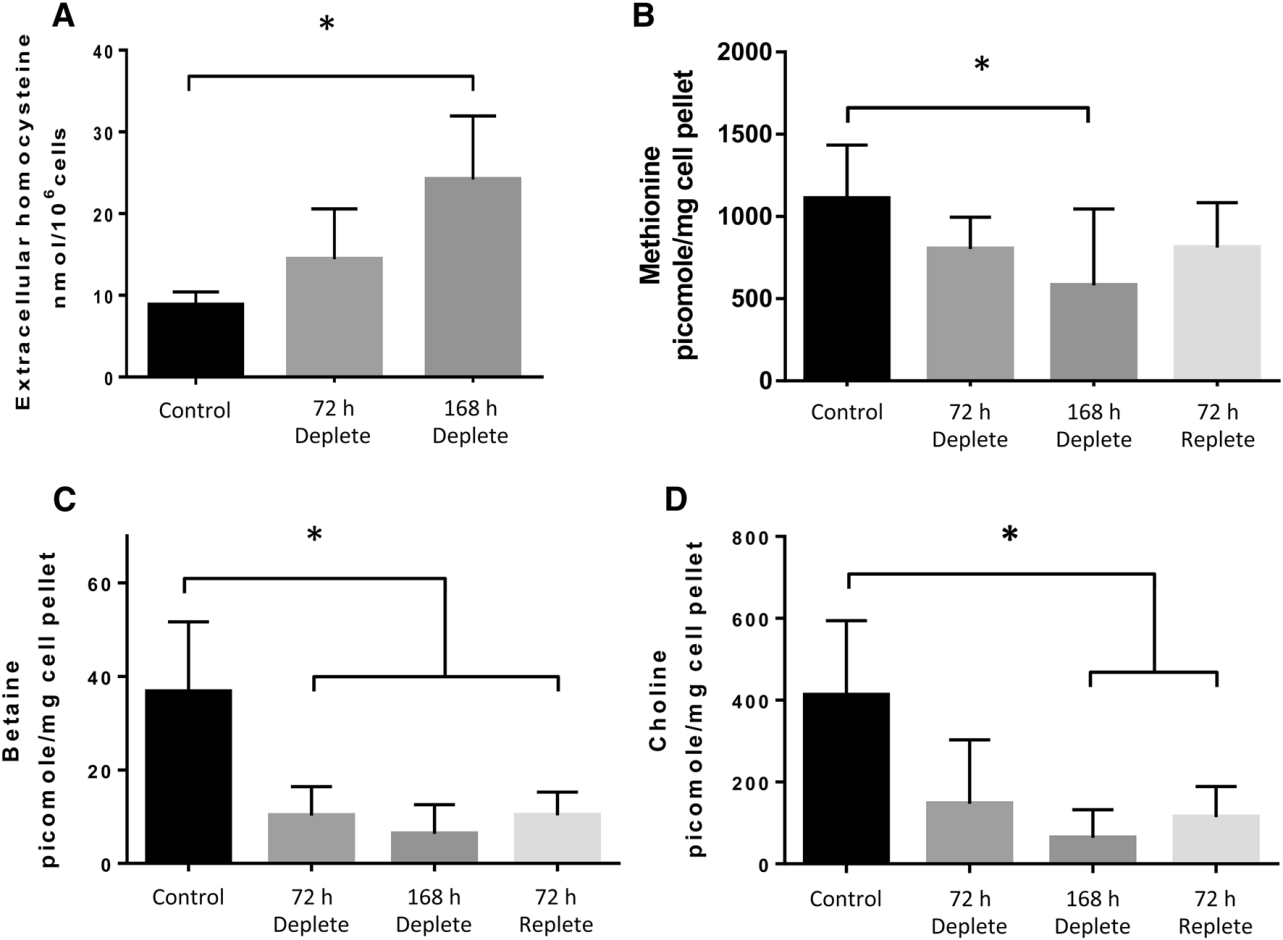
Validation of methyl donor depletion in UD-SCC2 cells. Disruption to the one carbon metabolism cycle leads to an increase in extracellular homocysteine and loss of intracellular methionine, choline, and betaine. Extracellular homocysteine concentration was measured by HPLC and normalised to cell number. **a** Concentration of extracellular homocysteine increased in UD-SCC2 cells following 72 and 168 h culture in depleted (5%) methyl donor medium compared to control cells (100%). The intracellular levels of **b** methionine, **c** betaine, and **d** choline, measured by LC-MS/MS were all significantly decreased in UD-SCC2 cells upon methyl donor depletion compared to controls (*p < 0.05) and showed signs of recovery after repletion in 100% methyl donor culture conditions for 72 h. *n* = 3 independent experiments performed in triplicate; one-way independent ANOVA with a Bonferroni post-hoc comparison

**Fig. 2:**
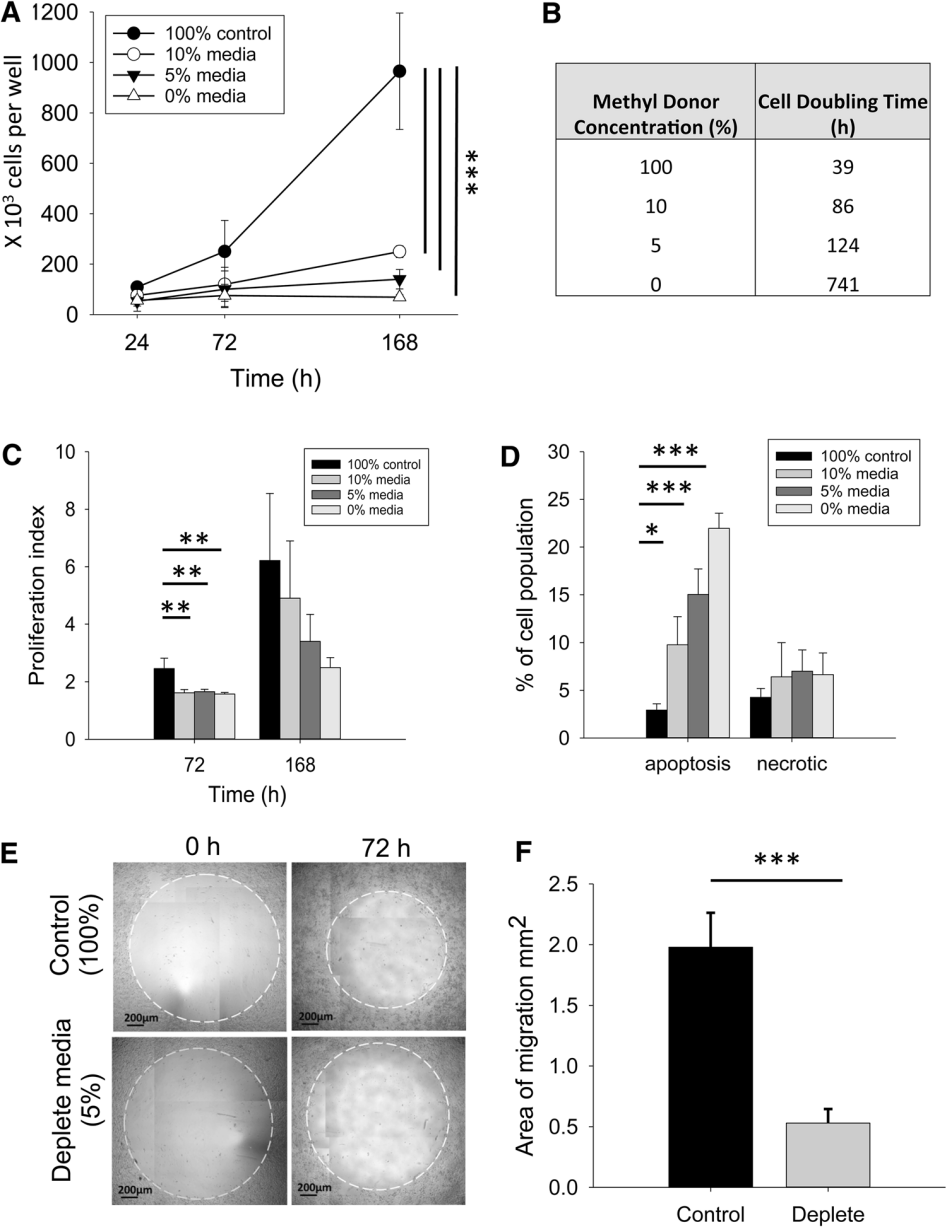
Effect of methyl donor depletion on UD-SCC2 cell growth, viability, and migration. **a** Change in cell number for cells cultured in media containing three different levels of methyl donor depletion (0, 5 and 10%) compared to cells grown in control media (100%). **b** Cell doubling time for UD-SCC2 cells cultured in media containing three different levels of methyl donor depletion (10, 5 and 0%) compared to cells grown in control media (100%). **c** Proliferation of cells cultured in media containing three different levels of methyl donor depletion (0, 5, and 10%), compared to cells grown in control media (100%) for 72 and 168 h. Higher proliferation index indicates more proliferating cells and increased proliferation rates. **d** Proportion of cells undergoing apoptosis (Annexin V positive) or necrosis (PI positive, Annexin V negative) following 168 h depletion (5% depletion; *n* = 4). **e** Cell migration into an exclusion zone (*white dotted line* on representative images) following 72 h depletion was compared to cells grown in complete media (100%). All cells were treated with mitomycin C prior to use in the migration assay to inhibit cell proliferation. **f** Area of migration quantified from three independent experiments (independent Student’s *t* test). One-way independent ANOVA with Bonferroni post-hoc comparison; *p < 0.05, **p < 0.01, **p < 0.005

**Fig. 3:**
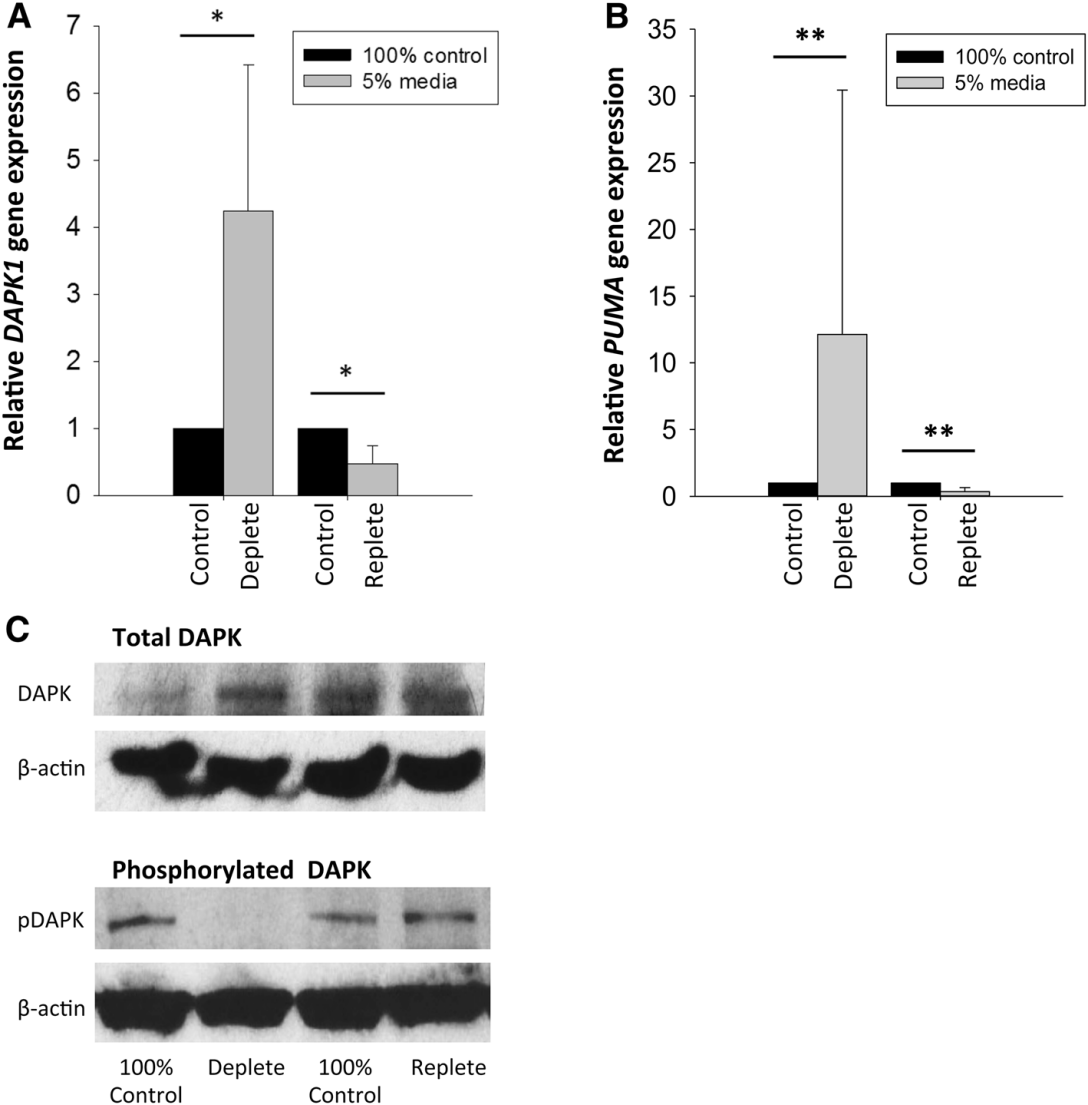
Effect of methyl donor depletion on *DAPK1* gene expression and protein levels. Gene expression of **a** *DAPK1* and **b** *PUMA* in UD-SCC2 cells measured by qPCR relative to β2-microglobulin expression after 168 h depletion and 72 h repletion. **c** Protein expression of total DAPK1 protein and phosphorylated DAPK1 (160 kDa) along with β-actin loading control (45 kDa) in UD-SCC2 cells. In **a**–**c**, gene and protein expression was measured in UD-SCC2 cells following methyl donor depletion for 168 h with medium containing 5% methyl donors (deplete) followed by a further 72 h culture in medium containing 100% methyl donors (replete). Control cells were continuously cultured in 100% methyl donor containing medium alongside deplete and replete cells

**Fig. 4:**
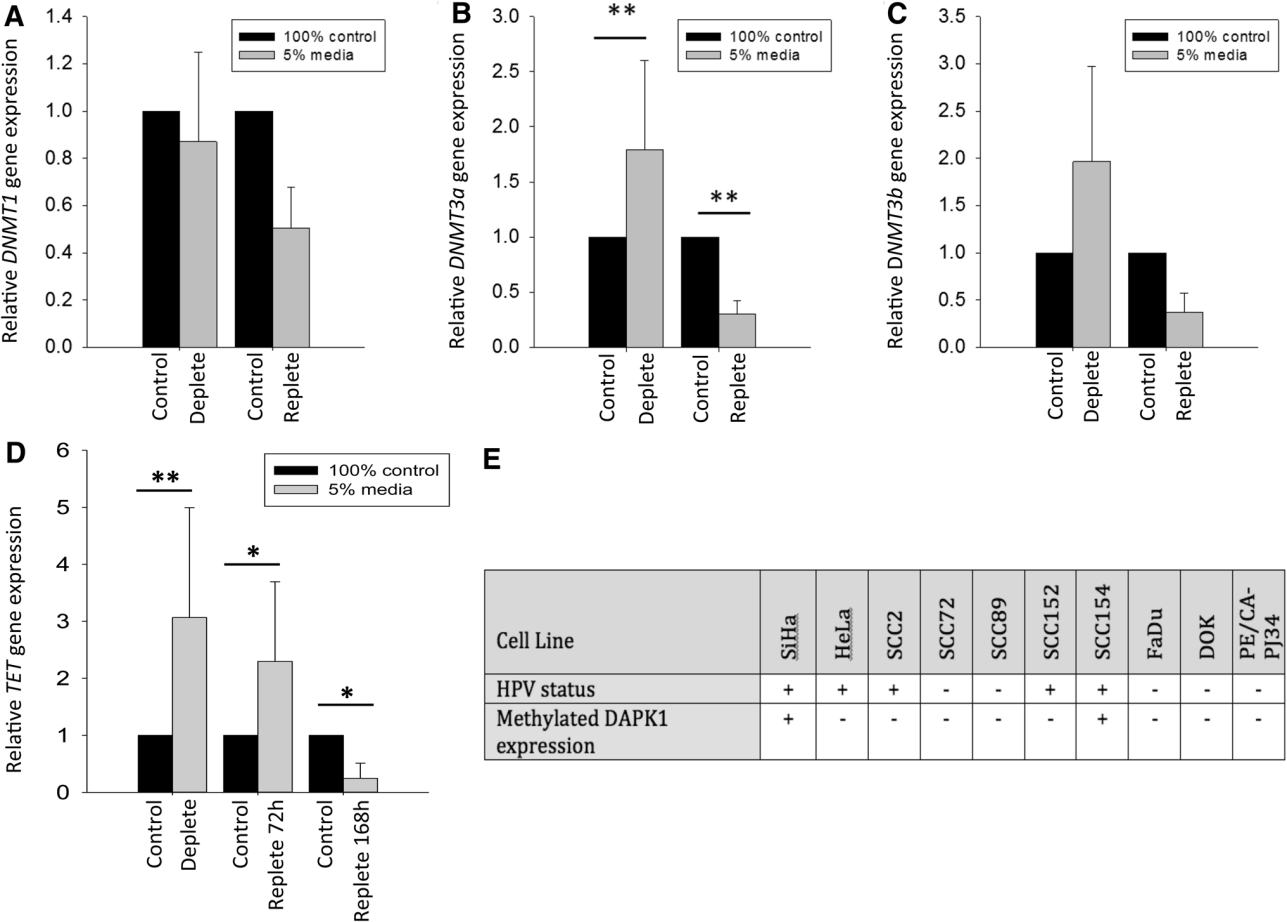
Effect of methyl donor depletion and repletion on DNMT and TET1 expression. Gene expression of *DNMT1* (**a**), *DNMT3a* (**b**), and *DNMT3b* (**c**) and **d** TET1 relative to β2 M expression was measured using qPCR in UD-SCC2 cells. Expression was measured following methyl donor depletion for 168 h with medium containing 5% methyl donors (deplete) followed by a further 72 h culture in medium containing 100% methyl donors (and 168 h for TET1) (replete). Control cells were continuously cultured in 100% methyl donor containing medium alongside deplete and replete cells. *n* = 6, independent Student’s *t* test, **p < 0.01. **e** Presence of methylated *DAPK1* promoter in a panel of HNSCC cell lines as measured by qMSP; the cervical cell lines SiHa were used as controls. Table shows cells positive for *DAPK1* methylation (+, Ct values <35) and cells negative for methylated *DAPK1* (−, Ct values >35). All cell lines tested were positive for β-actin (mean Ct value 25 ± 0.47)

Statistical analysis was performed using SPSSv.20 (IBM, Hampshire, UK) and data are expressed as mean ± standard deviation. All data were tested for homogeneity of variance using Levene’s prior to parametric statistical analysis. Pairwise comparisons were conducted using an Independent Student’s unpaired *t* test (Figs. 1, 2, 3b, 4a) and group-wise comparisons were carried out using one-way independent ANOVA with Bonferroni post-hoc comparison (Fig. 3c–e). Differences were considered significant if *p* < 0.05.

## Results

### Establishment and validation of an in vitro model of methyl donor depletion

Disturbance to the methyl cycle caused by a deficiency of methyl donors is characterised by an increase in homocysteine concentration; this is well documented as an increase in plasma homocysteine in human studies [36] and in extracellular homocysteine levels in in vitro studies [37]. Figure 1a shows an increase in the extracellular homocysteine of UD-SCC2 after 72 h of growth in 5% methyl donor-deficient medium compared with growth in complete medium, and by 168 h, the difference reached statistical significance (*p* < 0.05). Unlike the UD-SCC2 cell line, the UPCI-SCC72 cells were unable to proliferate and survive in culture media containing methyl donor concentrations below 20% of standard cell culture concentrations. In UPCI-SCC72 cells, an increase in extracellular homocysteine was observed at 72 and 168 h of growth in 20% methyl donor medium (Supplementary Fig. 2A). These data confirm a functional disturbance to the methyl cycle in both UD-SCC2 and UPCI-SCC72 cells, validating the methyl donor depletion model. In addition, for UD-SCC2 cells, the mean intracellular concentration of methionine, choline, and betaine was significantly (*p* < 0.05) decreased by 50, 90, and 85%, respectively after 168 h in methyl donor deplete (5%) medium compared to those cultured in control medium (100%) (Fig. 1b–d). After 72 h in replete conditions (100% medium), the intracellular concentrations of methionine, choline, and betaine started to increase toward control cell concentrations, although these levels did not reach statistical significance compared to 168 h depleted cells (Fig. 1b–d).

### Methyl donor depletion alters the phenotype of HNSCC cells

A reduction in methyl donor status in UD-SCC2 cells led to a significant (*p* < 0.001) dose-dependent decrease in cell number compared to 100% control medium over 168 h (Fig. 2a). Similar dose-dependent findings were observed for UPCI-SCC72 cells (Supplementary Fig. 2B), although we found these cells to be less tolerant of methyl donor depletion, with concentrations below 20% causing cell death. In addition, UD-SCC2 cells grown in complete medium (100%) displayed a cell-doubling time of 39 h, whereas cells cultured with 10, 5, and 0% of complete methyl donor concentrations displayed substantially longer cell-doubling times of 86, 124, and 741 h, respectively (Fig. 2b). The same trend was observed for UPCI-SCC72 cells (Supplementary Fig. 2C). The reduction in UD-SCC2 cell-doubling time was, in part, a result of decreased cell proliferation. The proliferation index of deplete cells measured using flow cytometry was significantly (*p* < 0.005) lower than that for control cells after 72 h and dose-dependent after 168 h (Fig. 2c). Because change in cell number is affected by both cell proliferation and cell death, we measured cell apoptosis and necrosis after 168 h depletion. The proportion of cells undergoing apoptosis increased significantly in a dose-dependent manner when cultured with reducing levels of methyl donors in both UD-SCC2 (Fig. 2d) and UPCI-SCC72 cells (Supplementary Fig. 2D). Approximately, 15% of UD-SCC2 cells cultured in methyl donor depleted conditions (5%) were apoptotic, whereas less than 3% of the cells cultured in medium containing 100% methyl donors were apoptotic after 168 h (Fig. 2d). The proportion of necrotic cells was not affected by methyl donor depletion (Fig. 2d). UD-SCC2 cells cultured in methyl donor depleted medium (5%) displayed visibly reduced cell motility after 72 h (Fig. 2e, f). Image analyses showed that cell migration was fourfold lower in methyl donor deplete cells compared to those cultured in complete medium (*p* < 0.001; Fig. 2f). This observed difference was due to cell migration and was not a consequence of cell proliferation, because cells were pre-treated with mitomycin C to inhibit cell division prior to the migration assay. The reduced capacity for cell migration and proliferation was not due to metabolic shock in methyl depleted cells as their metabolism, as determined by MTT assay, was not significantly different from control cells (Supplementary Fig. 3a, b).

### Methyl donor depletion increases *DAPK1* and *PUMA* expression and affects *DAPK1* phosphorylation status in UD-SCC2 cells

Since methyl donor depletion caused significantly more apoptosis in HPV-positive UD-SCC2 cells compared to HPV-negative UPCI-SCC72 cells, we further examined UD-SCC2 cells for likely mechanisms of increased methyl-donor deplete-induced apoptosis. Gene expression of *DAPK1* and *PUMA*, key enzymes involved in mediating apoptosis and autophagy, was increased fourfold (*p* < 0.05) and 13-fold (*p* < 0.01), respectively, following culture in methyl donor deplete medium (5%) for 168 h compared to expression in cells cultured in methyl donor containing (100%) medium (Fig. 3a, b). This change in expression was completely reversed when cells were cultured in replete (100%) methyl donor medium for a further 72 h (Fig. 3a, b). Levels of total DAPK1 protein expression as well as the phosphorylated, inactive form were measured by immunoblotting. In support of the qPCR data, levels of total DAPK1 protein increased upon methyl donor depletion (5%) compared to cells cultured in 100% medium (Fig. 3c). In addition, an immunoblot band corresponding to the phosphorylated, inactive form of DAPK1 was observed in extracts derived from cells cultured in methyl donor containing medium (100%), whereas phosphorylated DAPK1 (pDAPK1) was absent in lysates from cells cultured in deplete conditions (5%). When the cells were returned to control media, the level of pDAPK1 returned to control levels demonstrating the effect was reversible (Fig. 3c).

### Expression of *DNMT 3a* and TET1 is increased in methyl donor deplete HPV-positive HNSCC cells

Expression of *DNMT1*, the enzyme responsible for maintaining DNA methylation, was not altered in UD-SCC2 cells depleted of methyl donors (5%) for 168 h when compared to cells cultured in complete (100%) medium, or following culture in replete medium for a further 72 h (Fig. 4a). However, expression of *DNMT3a*, the enzyme responsible for de novo DNA methylation, was significantly (*p* < 0.01) increased following methyl donor depletion for 168 h and this change was reversed when these cells were cultured with methyl donor-containing medium, *p* < 0.01 (Fig. 4b). A similar trend was also observed for expression of *DNMT3b*, although this was not statistically significant (Fig. 4c). In addition, expression of *TET1* was significantly (*p* < 0.01) increased in UD-SCC2 cells depleted of methyl donors (5%) for 168 h when compared to cells cultured in complete (100%) medium. Unlike expression of *DNMT3a*, expression of TET1 remained elevated even after 72 h culture in replete (100% methyl donor-containing) medium, but expression was reversed after 168 h in replete culture conditions (Fig. 3d).

### The promoter of DAPK1 is not consistently methylated in HPV-positive HNSCC cells

Since increased *DAPK1* gene methylation has been associated with persistent HPV infection [38] and the promoter region of *DAPK1* has also been shown to be hypermethylated in HNSCC [18], we investigated the methylation status of the *DAPK1* promoter in a panel of six HNSCC cell lines, an oral dysplastic cell line, and a basaloid squamous cell carcinoma cell line (all cultured in methyl donor containing medium). SiHa cervical carcinoma cells were used as methylated *DAPK1* promoter controls. Of all the HNSCC and dysplastic cell lines tested, only SCC154 displayed a methylated *DAPK1* promoter (Fig. 4e).

## Discussion

There is evidence to suggest that altered folate status influences the risk of cancer and there are plausible mechanisms to support this [39, 40]. Studies of methyl donors other than folate have indicated a complex interaction between methyl donor availability and function, whereby deficiency in one methyl donor may be compensated for by another, to maintain a functional methyl cycle [41, 42]. However, very few studies have examined effects of depletion of more than a single methyl donor, where the opportunity for such compensation is minimised. In this study, we successfully developed a model of functional methyl donor deficiency that was characterised by an increased concentration of homocysteine in the extracellular medium and decreased intracellular levels of methyl donors, indicating disturbance to the methyl cycle [36, 37]. The time-related change in extracellular homocysteine was different in the two cell lines used. This may reflect cell-specific sensitivity to methyl donor depletion, including differential up-regulation of folate receptors and/or homocysteine metabolism [43, 44].

Cancer cell motility is important for tumour cell invasion during HNSCC progression and subsequently leads to the development of local or distant site metastases. Our data demonstrate for the first time that HNSCC cell migration is significantly impaired in methyl donor depleted conditions. Moreover, the cellular response is unlikely to be due to metabolic shock as the metabolic status of deplete and control cells was similar. This is an important finding as increased cell migration has been associated with poor prognosis in HNSCC [45] and the ability to immobilise cancer cells is an important target for cancer drug development. There has been relatively little research into the effects of methyl donor depletion on tumour cell migration and findings suggest that effects may be cell specific. Human HCT116 colon carcinoma cells showed increased migration in folate-deplete conditions [46], whereas Graziosi et al. reported reduced cell migration in methionine-depleted gastric cancer cells [47]. The effects on migration may be mediated by epigenetic alterations in the expression of genes important for cell locomotion. The actin cytoskeleton plays a central role in cell movement and studies on the effects of methyl donor depletion in various cell types suggest that genes associated with the regulation of the actin cytoskeleton are invariably down regulated. Duthie et al. reported reduced expression of proteins important for cytoskeleton organization in folate-depleted human colonocytes [8], whilst Jhaveri et al. showed down-regulation of cytoskeleton 14 in folate-depleted human KB (HeLa) cells [48].

The observed reduction in cell proliferation in methyl-donor deplete UD-SCC2 cells could, in part, be explained by a shortage of purine and pyrimidines that are essential for DNA production, as methyl donors are required for the synthesis of these molecules. An alternative explanation may be that methyl donor-depleted cells are arrested at G1 in the cell cycle, as has previously been reported in human and murine B cells with reduced levels of methionine and SAM [49]. The effect on cell proliferation observed in this study was dose-dependent and the rate of cell proliferation was restored following repletion. The decrease in cell proliferation rate detected in methyl donor-deficient cultures did not account entirely for the reduction in cell number observed. Cell death, through apoptosis, also increased in the methyl donor depleted conditions, while cell necrosis remained unchanged.

The role that folate plays in DNA synthesis and cell division is well understood and this is the basis for the use of anti-folate drugs in cancer treatment. A decrease in methyl donor availability is expected to lead to a fall in cell proliferation and this is what we observed. Evidence linking folate status with apoptosis is limited and inconsistent [50–52]. An increase in apoptosis is understood to have beneficial effects in cancer and a low level of apoptosis in HNSCC is associated with poor prognosis [53]. Low availability of methyl donors, in particular folate, may lead to reduced DNA synthesis and thus inhibit tumour cell proliferation of established tumours.

*DAPK1* and *PUMA* are pro-apoptotic genes known to be up-regulated when cells encounter stress [54, 55]. We hypothesised that activation of pro-apoptotic genes mediates the increase in apoptosis observed in methyl donor depleted cells. *DAPK1* and *PUMA* mRNA levels significantly increased following methyl donor depletion and, in the case of DAPK1, this was associated with an increase in protein levels as well, a response that was reversed upon methyl donor repletion. Interestingly, cells that were methyl donor deficient also displayed reduced levels of phosphorylated DAPK1, the inactive form of the protein, compared to cells cultured in complete media. These data suggest that upon prolonged methyl donor depletion, cells respond by not only increasing the gene expression of *DAPK1* but also by increasing the active, non-phosphorylated form of the protein that then drives the pro-apoptotic pathway leading to the programmed cell death of HNSCC cells.

A functionally impaired methyl cycle was associated with effects on the expression of DNMTs and TET1. The expression of *DNMT1*, the DNMT involved in maintaining DNA methylation status following cell division [19], was not influenced by a reduction in methyl donor status in UD-SCC2 cells. Reports of effects of folate depletion on DNMT1 expression in cultured cells are inconsistent; Stempak et al., [56] reported a down-regulation in mouse fibroblasts and human colon cancer cells, whilst Hayashi et al. [32] observed an up-regulation in response to folate depletion of the same human colon cancer cells. In contrast to *DNMT1*, expression of *DNMT3a* was increased in methyl donor deplete UD-SCC2 cells and this effect was reversed upon methyl donor repletion. A similar direction of change was seen for *DNMT3b*, although the effect was not statistically significant. Others have reported an increase in the expression of *DNMT3a* in response to methyl donor depletion in animal models and the relative resistance of *DNMT3b* to methyl donor depletion has also been documented in these studies [57, 58]. However, colon cancer cells depleted of folate alone are reported to show a decrease in *DNMT3a* [32, 56], and Poomipark et al. reported a decrease in *DNMT3a* and *DNMT3b* in a folate and methionine-depleted cervical cancer cell line C4II [22], adding to the evident inconsistency of response to single or multiple methyl donor depletion in different experimental systems. The fact that we were able to demonstrate reversibility of the effect on *DNMT3a* expression is a strong indicator of the causal relationship between methyl donor availability and *DNMT3a* expression in HNSCC cells. It is plausible that in methyl donor deplete HNSCC cells, expression of *DNMT3a* and *TET1* (responsible for DNA demethylation) is increased as an adaptive response, to scavenge cellular sources of methyl groups and maintain epigenetic control of gene transcription. Deletion of *DNMT3a* has been shown to promote lung tumour progression [59] and expression has been shown to be higher in leukaemia compared with control cells [60]. Sun et al. recently showed that inhibition of *DNMT3a* in cervical cancer cells induced apoptosis, suggesting a cancer promoting effect of *DNMT3a* [61]. In our study, increased expression of *DNMT3a* and *TET1* was associated with increased apoptosis; whether these responses to methyl donor depletion are causally linked is not clear.

Methylation of both host and viral DNA is thought to be important in HPV-positive cancers, with distinct methylation patterns observed in HPV-positive and negative disease [62]. It has previously been shown that the *DAPK1* promoter is hyper-methylated in oral cancer cells and the prevalence of *DAPK1* methylation in oral cancer ranges from 7 to 68% [18]. This led us to hypothesise that in methyl donor deplete conditions, the lack of readily available methyl groups would result in loss of methylation within the *DAPK1* promoter, leading to increased *DAPK1* gene expression, increased protein production and activity, and, therefore, increased apoptosis. The previous studies have shown variations in gene methylation patterns following folate depletion in vitro depending on the cell type and gene of interest [39]. However, we found that the *DAPK1* promoter was not methylated in UD-SCC2 HPV-positive cells or in several other HNSCC/dysplastic (HPV-negative) cell lines. This was not due to a lack of specificity of the PCR primers used as SiHa cervical carcinoma cells displayed methylated *DAPK1* as previously described [63]. These data suggest that other mechanisms regulating *DAPK1* gene expression and activation are at play in these cells or that the *DAPK1* gene is under epigenetic control at other sites within the promoter region that were not covered by our primer sequences.

The evidence presented from this study indicates that methyl donor depletion of HNSCC cell lines results in a less aggressive cancer cell phenotype. Cancer cells which display reduced proliferation, increased apoptosis, and decreased migration are highly likely to produce less aggressive tumours *in vivo*. While low folate consumption is considered to be a risk factor for cancer at various sites, this may not be true for established cancers or in cells that contain microscopic neoplastic foci where folate could promote rather than prevent carcinogenesis through the provision of nucleotide precursors required for growth of rapidly dividing cancer cells [64, 65]. Indeed, there is increasing evidence from studies in animals and humans to suggest that whilst adequate folate status may be protective against cancer initiation, folate deficiency can slow cancer growth, whilst high intakes may enhance growth of certain cancers, including colorectal, breast, and prostate cancer [64–66]. Recent evidence extends this observation to other nutrients involved in the methyl cycle [67].

This study has demonstrated that depleting HNSCC cells of methyl donors has beneficial effects in terms of the cancer phenotype and provides mechanistic data to support evidence suggesting that high intakes of methyl donors may have adverse effects in established cancers or where pre-cancerous lesions exist.

## Electronic supplementary material

Below is the link to the electronic supplementary material.

Supplementary material 1 (PDF 289 KB)

## References

[1] Ferlay J, Soerjomataram I, Dikshit R, Eser S, Mathers C, Rebelo M, Parkin DM, Forman D, Bray F (2012) GLOBOCAN 2012 v1.0, Cancer Incidence and Mortality Worldwide: IARC CancerBase No. 11. Available via NLM. 2016 10.1002/ijc.29210 25220842

[2] Gatta G, Botta L, Sánchez MJ, Anderson LA, Pierannunzio D, Licitra L, Group, EW (2015). Prognoses and improvement for head and neck cancers diagnosed in Europe in early 2000s: the EUROCARE-5 population-based study. Eur J Cancer.

[3] Napier SS, Speight PM (2008). Natural history of potentially malignant oral lesions and conditions: an overview of the literature. J Oral Pathol Med.

[4] Kreimer AR, Clifford GM, Boyle P, Franceschi S (2005). Human papillomavirus types in head and neck squamous cell carcinomas worldwide: a systematic review. Cancer Epidemiol Biomark Prev.

[5] Chaturvedi AK, Engels EA, Pfeiffer RM, Hernandez BY, Xiao W, Kim E, Jiang B, Goodman MT, Sibug-Saber M, Cozen W, Liu L, Lynch CF, Wentzensen N, Jordan RC, Altekruse S, Anderson WF, Rosenberg PS, Gillison ML (2011). Human papillomavirus and rising oropharyngeal cancer incidence in the United States. J Clin Oncol.

[6] Blount BC, Mack MM, Wehr CM, MacGregor JT, Hiatt RA, Wang G, Wickramasinghe SN, Everson RB, Ames BN (1997). Folate deficiency causes uracil misincorporation into human DNA and chromosome breakage: implications for cancer and neuronal damage. Proc Nat Acad Sci USA.

[7] Titenko-Holland N, Jacob RA, Shang N, Balaraman A, Smith MT (1998). Micronuclei in lymphocytes and exfoliated buccal cells of postmenopausal women with dietary changes in folate. Mutat Res.

[8] Duthie SJ, Mavrommatis Y, Rucklidge G, Reid M, Duncan G, Moyer MP, Pirie LP, Bestwick CS (2008). The response of human colonocytes to folate deficiency in vitro: functional and proteomic analyses. J Proteome Res.

[9] Freedman ND, Park Y, Subar AF, Hollenbeck AR, Leitzmann MF, Schatzkin A, Abnet CC (2008). Fruit and vegetable intake and head and neck cancer risk in a large United States prospective cohort study. Int J Cancer.

[10] Kune GA, Kune S, Field B, Watson LF, Cleland H, Merenstein D, Vitetta L (1993). Oral and pharyngeal cancer, diet, smoking, alcohol, and serum vitamin A and beta-carotene levels: a case-control study in men. Nutr Cancer.

[11] Pelucchi C, Talamini R, Negri E, Levi F, Conti E, Franceschi S, La Vecchia C (2003). Folate intake and risk of oral and pharyngeal cancer. Ann Oncol.

[12] Petersen LF, Brockton NT, Bakkar A, Liu S, Wen J, Weljie AM, Bismar TA (2012). Elevated physiological levels of folic acid can increase in vitro growth and invasiveness of prostate cancer cells. BJU Int.

[13] Ly A, Lee H, Chen J, Sie KK, Renlund R, Medline A, Sohn KJ, Croxford R, Thompson LU, Kim YI (2011). Effect of maternal and postweaning folic acid supplementation on mammary tumor risk in the offspring. Cancer Res.

[14] Piyathilake CJ, Henao OL, Macaluso M, Cornwell PE, Meleth S, Heimburger DC, Partridge EE (2004). Folate is associated with the natural history of high-risk human papillomaviruses. Cancer Res.

[15] Flatley JE, McNeir K, Balasubramani L, Tidy J, Stuart EL, Young TA, Powers HJ (2009). Folate status and aberrant DNA methylation are associated with HPV infection and cervical pathogenesis. Cancer Epidemiol Biomark Prev.

[16] Xiao S, Tang YS, Khan RA, Zhang Y, Kusumanchi P, Stabler SP, Jayaram HN, Antony AC (2012). Influence of physiologic folate deficiency on human papillomavirus type 16 (HPV16)-harboring human keratinocytes in vitro and in vivo. J Biol Chem.

[17] Towle R, Truong D, Hogg K, Robinson WP, Poh CF, Garnis C (2013). Global analysis of DNA methylation changes during progression of oral cancer. Oral Oncol.

[18] Ha PK, Califano JA (2006). Promoter methylation and inactivation of tumour-suppressor genes in oral squamous-cell carcinoma. Lancet Oncol.

[19] Bestor T, Laudano A, Mattaliano R, Ingram V (1988). Cloning and sequencing of a cDNA encoding DNA methyltransferase of mouse cells. The carboxyl-terminal domain of the mammalian enzymes is related to bacterial restriction methyltransferases. J Mol Biol.

[20] Okano M, Bell DW, Haber DA, Li E (1999). DNA methyltransferases Dnmt3a and Dnmt3b are essential for de novo methylation and mammalian development. Cell.

[21] Ito S, Shen L, Dai Q, Wu SC, Collins LB, Swenberg JA, He C, Zhang Y (2011). Tet proteins can convert 5-methylcytosine to 5-formylcytosine and 5-carboxylcytosine. Science.

[22] Poomipark N, Flatley JE, Hill MH, Mangnall B, Azar E, Grabowski P, Powers HJ (2016). Methyl donor status influences DNMT expression and global DNA methylation in cervical cancer cells. Asian Pac J Cancer Prev.

[23] Lin Y, Hupp TR, Stevens C (2010). Death-associated protein kinase (DAPK) and signal transduction: additional roles beyond cell death. FEBS J.

[24] Altman BJ, Rathmell JC (2012). Metabolic stress in autophagy and cell death pathways. Cold Spring Harb Perspect Biol.

[25] Hoffmann TK, Sonkoly E, Hauser U, van Lierop A, Whiteside TL, Klussmann JP, Hafner D, Schuler P, Friebe-Hoffmann U, Scheckenbach K, Erjala K, Grénman R, Schipper J, Bier H, Balz V (2008). Alterations in the p53 pathway and their association with radio- and chemosensitivity in head and neck squamous cell carcinoma. Oral Oncol.

[26] White JS, Weissfeld JL, Ragin CC, Rossie KM, Martin CL, Shuster M, Ishwad CS, Law JC, Myers EN, Johnson JT, Gollin SM (2007). The influence of clinical and demographic risk factors on the establishment of head and neck squamous cell carcinoma cell lines. Oral Oncol.

[27] Rangan SR (1972). A new human cell line (FaDu) from a hypopharyngeal carcinoma. Cancer.

[28] Scherer WF, Syverton JT, Gey GO (1953). Studies on the propagation in vitro of poliomyelitis viruses. IV. Viral multiplication in a stable strain of human malignant epithelial cells (strain HeLa) derived from an epidermoid carcinoma of the cervix. J Exp Med.

[29] Friedl F, Kimura I, Osato T, Ito Y (1970). Studies on a new human cell line (SiHa) derived from carcinoma of uterus. I. Its establishment and morphology. Proc Soc Exp Biol Med.

[30] Chang SE, Foster S, Betts D, Marnock WE (1992). DOK, a cell line established from human dysplastic oral mucosa, shows a partially transformed non-malignant phenotype. Int J Cancer.

[31] Berndt A, Hyckel P, Könneker A, Katenkamp D, Kosmehl H (1997). Oral squamous cell carcinoma invasion is associated with a laminin-5 matrix re-organization but independent of basement membrane and hemidesmosome formation. Clues from an in vitro invasion model. Invasion Metastasis.

[32] Hayashi I, Sohn KJ, Stempak JM, Croxford R, Kim YI (2007). Folate deficiency induces cell-specific changes in the steady-state transcript levels of genes involved in folate metabolism and 1-carbon transfer reactions in human colonic epithelial cells. J Nutr.

[33] Friesen RW, Novak EM, Hasman D, Innis SM (2007). Relationship of dimethylglycine, choline, and betaine with oxoproline in plasma of pregnant women and their newborn infants. J Nutr.

[34] Balada E, Ordi-Ros J, Serrano-Acedo S, Martinez-Lostao L, Rosa-Leyva M, Vilardell-Tarres M (2008). Transcript levels of DNA methyltransferases DNMT1, DNMT3A and DNMT3B in CD4 + T cells from patients with systemic lupus erythematosus. Immunol.

[35] Nikolaidis G, Raji OY, Markopoulou S, Gosney JR, Bryan J, Warburton C, Walshaw M, Sheard J, Field JK, Liloglou T (2012). DNA methylation biomarkers offer improved diagnostic efficiency in lung cancer. Cancer Res.

[36] Moat SJ, Hill MH, McDowell IF, Pullin CH, Ashfield-Watt PA, Clark ZE, Whiting JM, Newcombe RG, Lewis MJ, Powers HJ (2003). Reduction in plasma total homocysteine through increasing folate intake in healthy individuals is not associated with changes in measures of antioxidant activity or oxidant damage. Eur J Clin Nutr.

[37] Nakano E, Taiwo FA, Nugent D, Griffiths HR, Aldred S, Paisi M, Kwok M, Bhatt P, Hill MH, Moat S, Powers HJ (2005). Downstream effects on human low density lipoprotein of homocysteine exported from endothelial cells in an in vitro system. J Lipid Res.

[38] Flatley JE, Sargent A, Kitchener HC, Russell JM, Powers HJ (2014). Tumour suppressor gene methylation and cervical cell folate concentration are determinants of high-risk human papillomavirus persistence: a nested case control study. BMC Cancer.

[39] Duthie SJ (2011). Folate and cancer: how DNA damage, repair and methylation impact on colon carcinogenesis. J Inherit Metab Dis.

[40] Ulrich CM, Potter JD (2007). Folate and cancer—timing is everything. JAMA.

[41] Niculescu MD, Zeisel SH (2002). Diet, methyl donors and DNA methylation: interactions between dietary folate, methionine and choline. J Nutr.

[42] Shin W, Yan J, Abratte CM, Vermeylen F, Caudill MA (2010). Choline intake exceeding current dietary recommendations preserves markers of cellular methylation in a genetic subgroup of folate-compromised men. J Nutr.

[43] Doucette MM, Stevens VL (2001). Folate receptor function is regulated in response to different cellular growth rates in cultured mammalian cells. J Nutr.

[44] Hultberg B (2003). Modulation of extracellular homocysteine concentration in human cell lines. Clin Chim Acta.

[45] Lyons AJ, Jones J (2007). Cell adhesion molecules, the extracellular matrix and oral squamous carcinoma. Int J Oral Maxillofac Surg.

[46] Wang TP, Hsu SH, Feng HC, Huang RF (2012). Folate deprivation enhances invasiveness of human colon cancer cells mediated by activation of sonic hedgehog signaling through promoter hypomethylation and cross action with transcription nuclear factor-kappa B pathway. Carcinogenesis.

[47] Graziosi L, Mencarelli A, Renga B, D’Amore C, Bruno A, Santorelli C, Cavazzoni E, Cantarella F, Rosati E, Donini A, Fiorucci S (2013). Epigenetic modulation by methionine deficiency attenuates the potential for gastric cancer cell dissemination. J Gastrointest Surg.

[48] Jhaveri MS, Wagner C, Trepel JB (2001). Impact of extracellular folate levels on global gene expression. Mol Pharmacol.

[49] Lin DW, Chung BP, Kaiser P (2014). S-adenosylmethionine limitation induces p38 mitogen-activated protein kinase and triggers cell cycle arrest in G1. J Cell Sci.

[50] Garcia-Crespo D, Knock E, Jabado N, Rozen R (2009). Intestinal neoplasia induced by low dietary folate is associated with altered tumor expression profiles and decreased apoptosis in mouse normal intestine. J Nutr.

[51] Cao DZ, Sun WH, Ou XL, Yu Q, Yu T, Zhang YZ, Wu ZY, Xue QP, Cheng YL (2005). Effects of folic acid on epithelial apoptosis and expression of Bcl-2 and p53 in premalignant gastric lesions. World J Gastroenterol.

[52] Hsu HC, Chiou JF, Wang YH, Chen CH, Mau SY, Ho CT, Chang PJ, Liu TZ (2013). Folate deficiency triggers an oxidative-nitrosative stress-mediated apoptotic cell death and impedes insulin biosynthesis in RINm5F pancreatic islet β-cells: relevant to the pathogenesis of diabetes. PLoS One.

[53] Xie X, Clausen OP, De Angelis P, Boysen M (1999). The prognostic value of spontaneous apoptosis, Bax, Bcl-2, and p53 in oral squamous cell carcinoma of the tongue. Cancer.

[54] Deiss LP, Feinstein E, Berissi H, Cohen O, Kimchi A (1995). Identification of a novel serine/threonine kinase and a novel 15-kD protein as potential mediators of the gamma interferon-induced cell death. Genes Dev.

[55] Nakano K, Vousden KH (2001). PUMA, a novel proapoptotic gene, is induced by p53. Mol Cell.

[56] Stempak JM, Sohn KJ, Chiang EP, Shane B, Kim YI (2005). Cell and stage of transformation-specific effects of folate deficiency on methionine cycle intermediates and DNA methylation in an in vitro model. Carcinogenesis.

[57] Ghoshal K, Li X, Datta J, Bai S, Pogribny I, Pogribny M, Huang Y, Young D, Jacob ST (2006). A folate- and methyl-deficient diet alters the expression of DNA methyltransferases and methyl CpG binding proteins involved in epigenetic gene silencing in livers of F344 rats. J Nutr.

[58] Kovacheva VP, Mellott TJ, Davison JM, Wagner N, Lopez-Coviella I, Schnitzler AC, Blusztajn JK (2007). Gestational choline deficiency causes global and Igf2 gene DNA hypermethylation by up-regulation of Dnmt1 expression. J Biol Chem.

[59] Gao Q, Steine EJ, Barrasa MI, Hockemeyer D, Pawlak M, Fu D, Reddy S, Bell GW, Jaenisch R (2011). Deletion of the de novo DNA methyltransferase Dnmt3a promotes lung tumor progression. Proc Natl Acad Sci USA.

[60] Mizuno S, Chijiwa T, Okamura T, Akashi K, Fukumaki Y, Niho Y, Sasaki H (2001). Expression of DNA methyltransferases DNMT1, 3 A, and 3B in normal hematopoiesis and in acute and chronic myelogenous leukemia. Blood.

[61] Sun J, Ji J, Huo G, Song Q, Zhang X (2015). miR-182 induces cervical cancer cell apoptosis through inhibiting the expression of DNMT3a. Int J Clin Exp Pathol.

[62] van Kempen PM, Noorlag R, Braunius WW, Stegeman I, Willems SM, Grolman W (2014). Differences in methylation profiles between HPV-positive and HPV-negative oropharynx squamous cell carcinoma: a systematic review. Epigenetics.

[63] Leung RC, Liu SS, Chan KY, Tam KF, Chan KL, Wong LC, Ngan HY (2008). Promoter methylation of death-associated protein kinase and its role in irradiation response in cervical cancer. Oncol Rep.

[64] Kim YI (2006). Folate: a magic bullet or a double edged sword for colorectal cancer prevention?. Gut.

[65] Bistulfi G, Foster BA, Karasik E, Gillard B, Miecznikowski J, Dhiman VK, Smiraglia DJ (2011). Dietary folate deficiency blocks prostate cancer progression in the TRAMP model. Cancer Prev Res (Phila).

[66] Deghan Manshadi S, Ishiguro L, Sohn KJ, Medline A, Renlund R, Croxford R, Kim YI (2014). Folic acid supplementation promotes mammary tumor progression in a rat model. PLoS One.

[67] Kadaveru K, Protiva P, Greenspan EJ, Kim YI, Rosenberg DW (2012). Dietary methyl donor depletion protects against intestinal tumorigenesis in Apc(Min/+) mice. Cancer Prev Res (Phila).

