# Supplementary material for: Methyl-donor depletion of head and neck cancer cells in vitro establishes a less aggressive tumour cell phenotype
Source: Eur J Nutr. 2017 Mar 1;57(4):1321–32. doi: 10.1007/s00394-017-1411-5 (PMC5959985; doi:10.1007/s00394-017-1411-5)
Supplement: Supplementary file 1 — Supplementary material 1 (PDF 289 KB) [file 394_2017_1411_MOESM1_ESM.pdf]

European Journal of Nutrition

Methyl-donor depletion of head and neck cancer cells *in vitro* establishes a less aggressive tumour cell phenotype

Vanessa Hearnden[1,2], Hilary J. Powers[1], Abeir Elmogassabi[1], Rosanna Lowe[1], Craig Murdoch[2*]

[1] Human Nutrition Unit, Department of Oncology, University of Sheffield, Sheffield, S10 2RX, UK

[2] School of Clinical Dentistry, University of Sheffield, Sheffield, S10 2TA, UK

* **Corresponding author**: Craig Murdoch; **E-mail**: c.murdoch@sheffield.ac.uk

Supplementary Figure 1

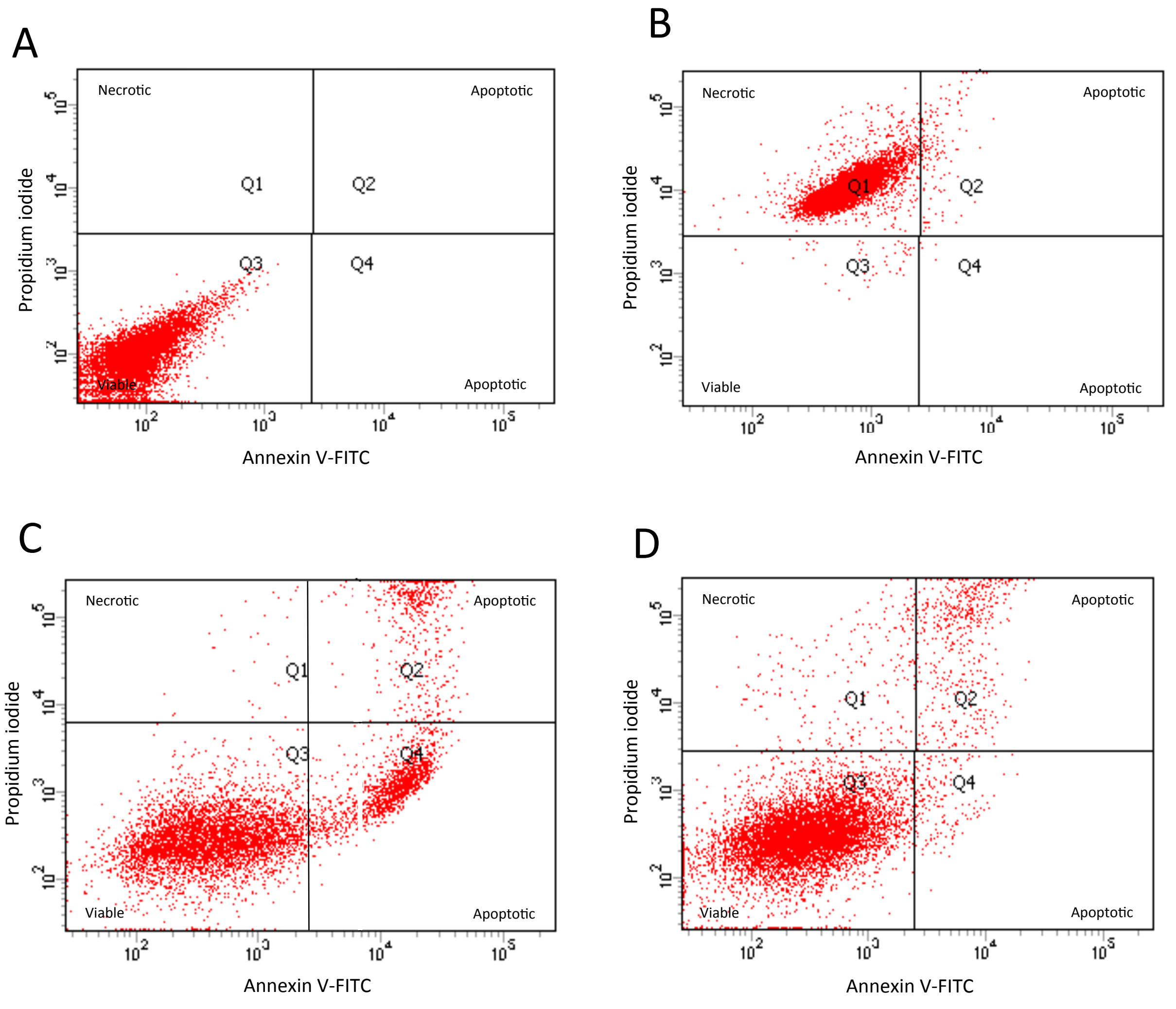

Supplementary Figure 1. Flow cytometric gating strategy used to select apoptotic and necrotic cell populations. UD-SCC2 or UPCI-SCC72 cells (red dots) cultured in complete or deplete methyl donor medium were stained with Annexin V-FITC and propidium iodide as described in the materials and methods and analysed by flow cytometry. (A) dot plot showing unstained, viable UD-SCC2 cells. (B) UD-SCC2 cells treated with 1% saponin in PBS as a positive control for necrosis (propidium iodide positive staining). (C) UD-SCC2 cells treated with camptothecin as a positive control for apoptosis (Annexin V-FITC positive staining). (D) UD-SCC2 cells cultured under 5 % methyl donor conditions and analysed for levels of apoptosis, necrosis and cell viability.

Supplementary Figure 2

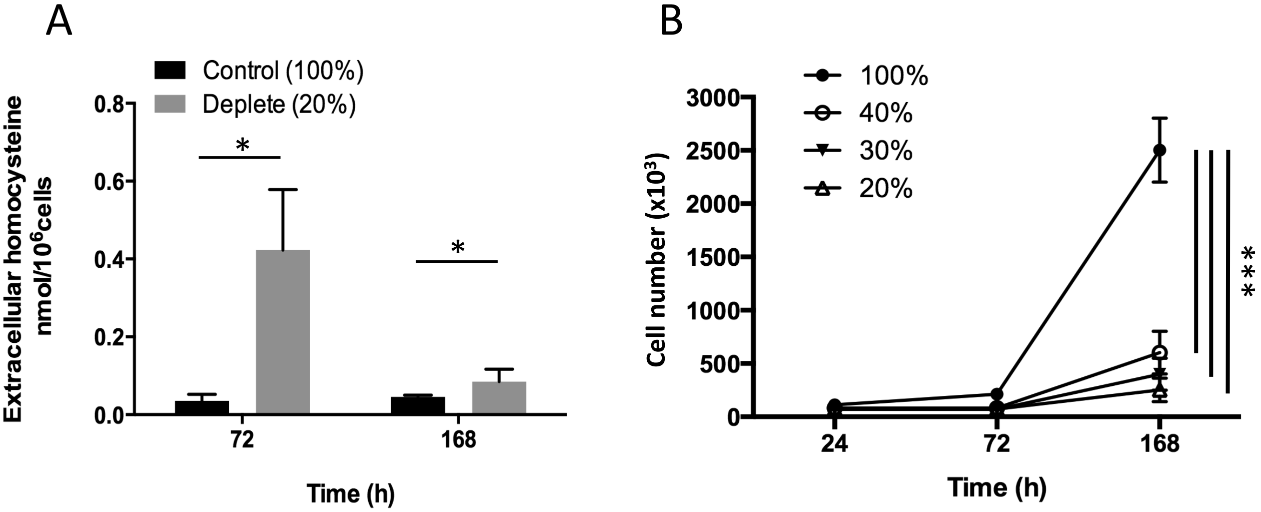

C

Methyl Donor Concentration (%)	Cell Doubling Time (h)
100	26.8
40	46.1
30	45.0
20	50.4

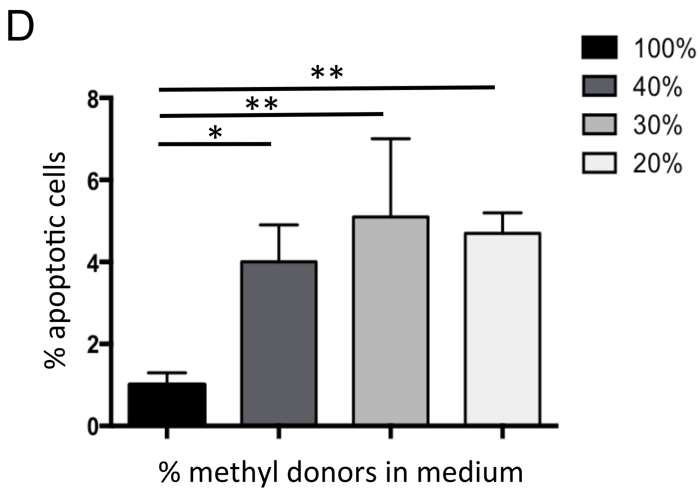

Supplementary Figure 2. Effect of methyl donor depletion on UPCI-SCC72 cell growth, doubling time and apoptosis. (A) Increased concentration of extracellular homocysteine in UPCI-SCC72 cells following 72 h and 168 h culture in depleted (20%) methyl donor medium compared to control cells (100%) Students-t test, *p<0.05 (B) Change in cell number and (C) cell doubling time for UPCI-SCC72 cells cultured in media containing three different levels of methyl donor depletion (20%, 30% and 40%) compared to cells grown in control media (100%). (D) Proportion of UPCI-SCC72 cells undergoing apoptosis (Annexin V-positive) following 168 h methyl donor depletion. All data are mean ± SD, n=3 independent experiments performed in triplicate. One-way independent ANOVA with Bonferonni post-hoc comparison.*p< 0.05, **p< 0.01, ***p< 0.001

Supplementary Figure 3

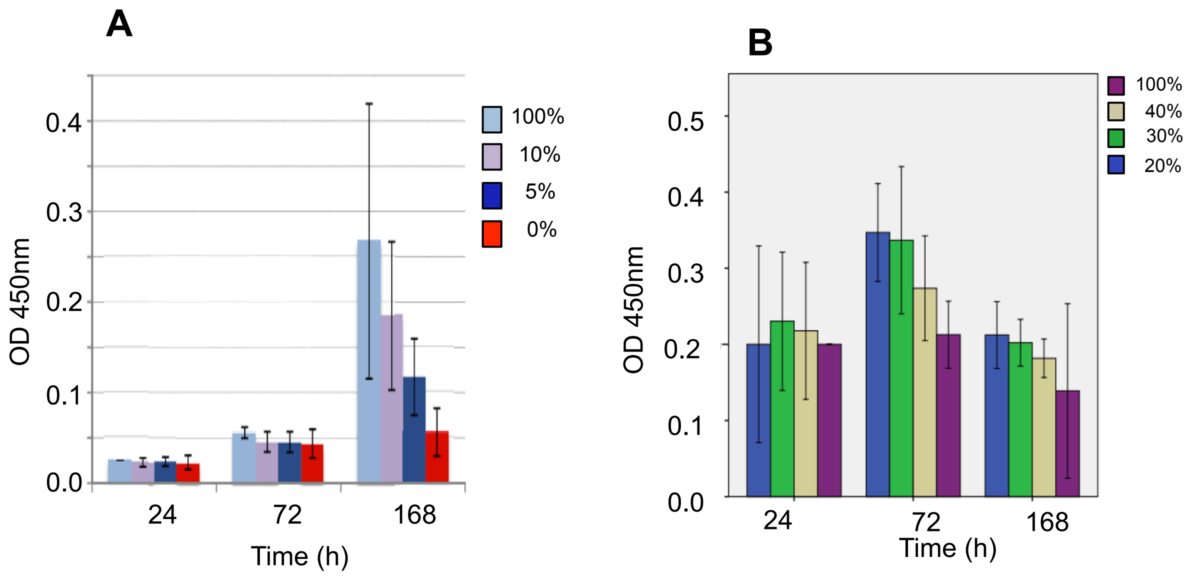

Supplementary Figure 3. **Effect of methyl donor depletion on cell metabolism.** No difference in the metabolic activity of UD-SCC2 (A) or UPCI-SCC72 (B) cells cultured in decreasing levels of methyl donors compared to cells grown in complete (100%) medium. The metabolic activity of cells was measured using MTT (3-(4,5-dimethylthiazol-2-yl)-2,5 diphenyl tetrazolium bromide). Cell monolayers were washed with PBS and MTT added for 40 minutes at 37°C, 5% CO_2. MTT solution was removed and the intracellular formazan salt solubilised by addition of acidified isopropanol. The optical density of released dye was measured at 540 nm with a reference 630 nm. Data are mean ± SD, n=3 independent experiments performed in triplicate.
